# Differences in Hemodynamic, Hormonal and Heart Rate Variability Parameters in Complication-Free Pregnancies Compared to Individuals with Gestational Diabetes Mellitus and Preeclampsia: An Observational Retrospective Analysis

**DOI:** 10.3390/life11070626

**Published:** 2021-06-29

**Authors:** Max L. Eckstein, Othmar Moser, Andreas Rössler, Manfred G. Moertl, Andreas Jantscher, Ilona Papousek, Johann Wagner, Karin Schmid-Zalaudek, Harald Sourij, Gerlies Treiber, Helmut K. Lackner

**Affiliations:** 1 Department of Sport Science, Division of Exercise Physiology and Metabolism, University of Bayreuth, 95447 Bayreuth, Germany; max.eckstein@uni-bayreuth.de; 2 Otto Loewi Research Center, Division of Physiology, Medical University of Graz, 8036 Graz, Austria; andreas.roessler@medunigraz.at (A.R.); an.jantscher@medunigraz.at (A.J.); johann.wagner@medunigraz.at (J.W.); karin.schmid@medunigraz.at (K.S.-Z.); helmut.lackner@medunigraz.at (H.K.L.); 3 Clinical Center Klagenfurt, Department of Obstetrics and Gynecology, 9020 Klagenfurt, Austria; manfred.moertl@medunigraz.at; 4 Department of Psychology, Biological Psychology Unit, University of Graz, 9020 Graz, Austria; ilona.papousek@uni-graz.at; 5 Department of Internal Medicine, Division of Endocrinology and Diabetology, Interdisciplinary Metabolic Medicine Trials Unit, Medical University of Graz, Auenbruggerplatz 2, 8036 Graz, Austria; ha.sourij@medunigraz.at (H.S.); gerlies.treiber@medunigraz.at (G.T.)

**Keywords:** gestational diabetes, heart rate variability, autonomic regulation, preeclampsia, testosterone

## Abstract

To investigate differences in hemodynamic, hormonal and heart rate variability parameters in women following complication-free pregnancies (healthy), preeclampsia and gestational diabetes mellitus (GDM) after giving childbirth. Data of 60 women (healthy: n = 29, age 32.7 ± 4.5 years, BMI 24.2 ± 4.3 kg/m^2^; preeclampsia: n = 16, age 35.3 ± 4.4 years, 28.5 ± 6.4 kg/m^2^; GDM, n = 15, age 32.3 ± 6.0 years, BMI 26.4 ± 6.2 kg/m^2^) were included. Two visits were conducted 16 and 48 weeks after giving childbirth. Hair samples were taken for analysis of cortisol and testosterone. ECG and blood pressure were recorded at each visit. Data were analyzed via RM-ANOVA and post-hoc testing (*p* ≤ 0.05). Heart rate increased from visit 1 to visit 2, whereas SDNN decreased (both *p* = 0.03). RMSSD showed an increased trend for groups (*p* = 0.06). Testosterone in the GDM group was significantly higher compared to the other groups (*p* = 0.002). Cortisol levels were significantly higher following post-hoc testing GDM was different compared to healthy individuals (*p* = 0.02). Hemodynamic changes from week 16 to week 48 did not differ between groups (*p* > 0.05). No differences between individuals with preeclampsia and healthy individuals were found for all hemodynamic parameters (*p* > 0.05). The study showed higher levels of chronic stress indicators in GDM measured via heart rate variability and cortisol compared to women with a history of preeclampsia and healthy women.

## 1. Introduction

Human pregnancy leads to alterations in maternal metabolism [1] that may lead to prenatal complications such as preeclampsia or gestational diabetes mellitus (GDM). While preeclampsia affects 2–8% of all pregnancies, the global leading cause of maternal and perinatal morbidity and mortality [2], the prevalence of GDM ranges between 5 and 39% depending on the cohort investigated and thus represents a public health burden [3,4,5].

Preeclampsia is manifested as hypertension with proteinuria affecting multiple organ systems in the second half of pregnancy, almost doubling the risk for a subsequent cardiovascular events [6]. Women with GDM develop a functional disorder marked by an increased insulin resistance with insufficient beta cell compensation leading to hyperglycaemia with features similar to type 2 diabetes (T2DM), often requiring glucose lowering treatment [7]. Following childbirth, the risk of hyperglycaemia is reduced while the risk of developing T2DM within 10 years following childbirth remains increased up to 50% [8]. Yet, it is unclear how the physiology of individuals with preeclampsia and GDM, respectively, is altered within 1 year after childbirth. Consequently, the aim of this study was to assess noninvasively hemodynamic markers, hormonal markers according to Slominski et al. [9] and heart rate variability (HRV) markers following childbirth in women with preeclampsia or GDM compared to women after complication-free pregnancies.

## 2. Materials and Methods

### 2.1. Setting and Study Population

This was a multi-center, retrospective outcome analysis performed in women after giving childbirth. The study protocol was approved by the local ethics committee (27-515 ex 14/15) and registered at the German Clinical Trials Register (drks.de; DRKS00024606).

The study was performed according to Good Clinical Practice and the Declaration of Helsinki. Prior to inclusion, participants received a detailed explanation of all study procedures by a medically trained researcher and subsequently gave their written informed consent. Eligibility criteria were singleton mothers between 18 and 40 years. For healthy women: uncomplicated singleton pregnancy with term delivery. For women with gestational diabetes: a fasting plasma glucose level of ≥5.6 mmol/L or a post 2-h oral glucose tolerance test plasma glucose level of ≥7.8 mmol/L or plasma glucose values exceeding the following criteria within 24th–28th week of gestation fasted ≥5.1 mmol/L, 1-h ≥ 10.0 mmol/L or 2-h ≥ 8.5 mmol/L. For women with preeclampsia: systolic blood pressure of ≥140 mmHg and/or diastolic blood pressure of ≥90 mmHg present at ≥20 week’s gestation in addition to proteinuria ≥300 mg per 24-h urine collection, protein/creatinine ratio ≥0.3. The definition of preeclampsia proteinuria was not necessary if any other of the following was present: a systolic blood pressure of ≥160 mmHg, diastolic blood pressure of 110 mmHg, impaired liver function, renal insufficiency, or pulmonary edema or symptoms involving neurological involvement such as headache or blurred vision. Participants were recruited postpartum and attended clinic for two visits at week 16 and week 48 after childbirth.

Continuous hemodynamic monitoring of blood pressure (BP) and heart rate (HR) was performed with the Task Force^®^ Monitor (TFM^®^; CNSystems, Graz, Styria, Austria). The Task Force^®^ Monitor provides all hemodynamic parameters completely synchronized. HR was recorded by 3-lead electrocardiography using CNSystems ECG-electrodes placed at the thoracic region. The continuous BP was derived from the finger using a refined version of the vascular unloading technique and corrected to absolute values with oscillometric BP measurement on the contralateral upper arm. The method is based on concentrically interlocking control loops for correct long-term tracing of finger BP and delivers, in contrast to intermittent set point re-adjustments of the conventional vascular unloading technique, BP without interruption. Parameters of HRV were resampled and computed via MATLAB^®^ (MathWorks, Natick, MA, USA) [10].

Hair samples were collected from the posterior vertex, cut as close to the scalp as possible and cut in pieces of 40 mm each. A minimum of 10 mg of hair was used for each sample. Extraction of cortisol was performed by overnight incubation with methanol. The supernatant was evaporated under nitrogen and re-suspended in phosphate-buffered saline (PBS) as conducted previously by Slominski et al. [9]. The cortisol and testosterone in the hair samples were subsequently measured using routinely used and commercially available salivary ELISA kit for cortisol (NovaTec Immundiagnostica GmbH, Dietzenbach, Hesse, Germany) and testosterone (Biozol Diagnostica GmbH, Eching, Bavaria, Germany).

### 2.2. Statistical Analysis

Analyses of variance were performed with visits 16 and 48 weeks after giving childbirth as the within-subjects factor group (healthy, preeclampsia and GMD) and as the between-subjects factor, and the respective cardiovascular, hormonal and heart rate variability variable as the dependent variable. Due to skewed distributions cortisol and testosterone values were natural logarithm transformed. To evaluate the difference between the three groups, Bonferroni corrected post-hoc tests were conducted.

## 3. Results

Data of 60 women (healthy: n = 29, age 32.7 ± 4.5 years, BMI 24.2 ± 4.3 kg/m^2^; preeclampsia: n = 16, age 35.3 ± 4.4 years, 28.5 ± 6.4 kg/m^2^; GDM, n = 15, age 32.3 ± 6.0 years, BMI 26.4 ± 6.2 kg/m^2^) were included. No significant differences were found for age (*p* = 0.16). BMI showed significant differences between groups (*p* = 0.05), based on significantly higher BMI in women with preeclampsia as compared to healthy women (*p* = 0.04). No significant differences were found for systolic blood pressure (SBP), mean arterial pressure (MAP) and diastolic blood pressure (DBP) (all *p* > 0.05). Heart rate was significantly different between visits (*p* = 0.028). Standard deviation of the NN interval (SDNN), a parameter of parasympathetic activity and major predictor of cardiac mortality, was significantly lower in individuals with GDM compared to individuals with preeclampsia (*p* < 0.001) and for visits between all groups (*p* = 0.028). Root mean the square root of the mean squared differences of successive NN intervals (RMSSD), a parameter of the balance between parasympathetic and sympathetic activity, was significantly different between all groups (*p* = 0.042). Cortisol levels were significantly different between uncomplicated pregnancies and individuals with preeclampsia (*p* < 0.001). Testosterone levels were significantly higher in individuals with preeclampsia compared to both other groups (*p* < 0.001). All results of this analysis are given in Table 1.

## 4. Discussion

To our knowledge, this is the first study investigating the effects of preeclampsia and GDM in comparison to uncomplicated pregnancies on hemodynamic parameters, hormonal levels and HRV after pregnancy. Our analysis showed no blood pressure differences between groups after 16 and 48 weeks postpartum. However, differences in heart rate, SDNN and RMSSD between groups and visits were found. Cortisol levels were higher in GDM compared to the control group, while testosterone levels remained increased in GDM compared to both other groups.

Villarreal et al. investigated sexual steroid levels of women with type 2 diabetes and GDM during pregnancy and found higher testosterone levels compared to controls [11]. The decreased estrogen levels and estradiol/testosterone ratio found in that particular study suggest a diminished aromatase activity during gestation in this particular cohort; however, it was yet to be known that testosterone levels remain increased in women with a history of GDM 16 to 48 weeks after giving childbirth. It is unclear how GDM may affect the perinatal programming towards an increased risk for chronic diseases, such as metabolic syndrome and type 2 diabetes in adulthood. The long-term effects on women’s health of significantly increased levels of testosterone and cortisol compared to the control group and women that had preeclampsia several weeks postpartum is yet to be investigated. However, previous studies have shown that increased testosterone levels in women are associated with insulin resistance, higher glucose concentrations and increased body fat—a precursor of developing metabolic syndrome more rapidly than women without a history of GDM [12].

Blood pressure levels are similar between all groups, which is coherent, since blood pressure values of preeclampsia are supposed to return back to normal within 12 weeks after childbirth [13]. Amaral et al. highlighted that preeclampsia may induce long-term consequences for vascular health postpartum [14]. This is not reflected by SBP or DBP. However, RMSSD in preeclampsia is the lowest and reflective of higher stress levels. Furthermore, SDNN, a marker predictive for cardiac mortality is significantly reduced up to 48 weeks postpartum in preeclampsia, which underpins the aforementioned potential long-term consequences [15]. Findings from this study are in line with previous findings from perinatal research [14,16]. Increased sleep deprivation in the first 16 weeks following childbirth may be considered as a possible confounding stress variable; however, this applies to all groups, which is why this factor is negligible. In future studies, sleep quality should be monitored to ensure that this parameter is not a confounder for hormonal measurements.

It has yet to be known that the effects of preeclampsia and GDM are still traceable for 48 weeks postpartum. A potential reason for the increased testosterone levels in the GDM group could be polycystic ovary syndrome (PCOS), which is associated with increased insulin insensitivity and higher androgen levels [17], though no cases with PCOS were included in our cohort. As a result of our study design, whether testosterone levels were increased prior to the manifestation of GDM in this cohort cannot be retraced. The course of androgen levels should be investigated during pregnancy and post-partum in future studies to further elucidate this process.

Our study is not without limitations since the number of visits and the sample sizes for each group are rather small and demand follow-up studies with a larger cohort to gain further insight into the physiological processes.

Furthermore, perinatal measurements were not conducted, though was not the aim of this study. Nonetheless, perinatal measurements would have been helpful to explain our findings in more detail since the course of the physiological adaptations in the perinatal phase could potentially give further information on how to predict physiological outcomes postpartum.

In addition, we did not conduct invasive methods to measure levels of sexual steroid hormones from venous blood, which is conducted in general practice. Using solely non-invasive methods to depict physiological alterations postpartum is novel and applicable since the applied techniques are easy to conduct and may also be applied in clinic and by other researchers.

## 5. Conclusions

In conclusion, our study highlights the long-term effects of preeclampsia and GDM up to 48 weeks after giving childbirth in comparison to healthy controls. These findings should stimulate further investigating of physiological alterations of the female body following pregnancy.

## Figures and Tables

**Table 1:** Anthropometric, cardiovascular and hormonal results.

	Healthy	Preeclampsia	GDM		*F*-Statistics
**n**	29	16	15		
SBP (mmHg)				group	*F*_(2,57)_ = 1.91; *p* = 0.158
Visit 1 (16 weeks)	106.8 ± 10.1	114.1 ± 12.4	107.5 ± 15.0	visit	*F*_(1,57)_ = 0.05; *p* = 0.819
Visit 2 (48 weeks)	106.3 ± 9.3	112.7 ± 14.1	108.4 ± 16.4	visit x group	*F*_(2,57)_ = 0.19; *p* = 0.827
MAP (mmHg)				group	*F*_(2,57)_ = 2.75; *p* = 0.073
Visit 1 (16 weeks)	84.4 ± 9.2	92.4 ± 10.9	86.1 ± 12.3	visit	*F*_(1,57)_ = 0.66; *p* = 0.421
Visit 2 (48 weeks)	84.2 ± 8.6	90.0 ± 10.2	85.7 ± 14.2	visit x group	*F*_(2,57)_ = 0.30; *p* = 0.739
DBP (mmHg)				group	*F*_(2,57)_ = 2.21; *p* = 0.120
Visit 1 (16 weeks)	68.4 ± 9.1	75.6 ± 10.4	70.0 ± 10.8	visit	*F*_(1,57)_ = 1.90; *p* = 0.174
Visit 2 (48 weeks)	68.5 ± 8.3	72.3 ± 8.5	68.2 ± 12.6	visit x group	*F*_(2,57)_ = 0.74; *p* = 0.481
Heart rate (bpm) ^*Visit*^				group	*F*_(2,57)_ = 0.07; *p* = 0.935
Visit 1 (16 weeks)	72.4 ± 8.0	72.8 ± 7.3	71.9 ± 9.6	visit	*F*_(1,57)_ = 5.08; *p* = 0.028
Visit 2 (48 weeks)	74.7 ± 10.0	73.8 ± 8.5	76.7 ± 13.0	visit x group	*F*_(2,57)_ = 0.75; *p* = 0.476
SDNN (ms) ^*Visit, GRP*^				group	*F*_(2,57)_ = 3.55; *p* = 0.035
Visit 1 (16 weeks)	49.1 ± 13.3	43.5 ± 13.2 ^3^	55.0 ± 18.7 ^2^	visit	*F*_(1,57)_ = 5.28; *p* = 0.025
Visit 2 (48 weeks)	42.4 ± 14.2	36.3 ± 12.9	51.9 ± 29.0	visit x group	*F*_(2,57)_ = 0.24; *p* = 0.788
RMSSD (ms) ^*GRP*^				group	*F*_(2,57)_ = 3.43; *p* = 0.042
Visit 1 (16 weeks)	38.9 ± 16.2	33.3 ± 14.3	49.6 ± 27.9	visit	*F*_(1,57)_ = 1.80; *p* = 0.184
Visit 2 (48 weeks)	31.4 ± 12.7	31.2 ± 18.5	47.4 ± 40.9	visit x group	*F*_(2,57)_ = 0.45; *p* = 0.639
Cortisol (pg/mg hair) ^*GRP*^				group	*F*_(2,57)_ = 7.31; *p* = 0.022
Visit 1 (16 weeks)	13.71 ± 6.25 ^3^	14.37 ± 7.54	18.81 ± 9.61 ^1^	visit	*F*_(1,57)_ = 1.37; *p* = 0.247
Visit 2 (48 weeks)	12.29 ± 5.11	14.02 ± 8.39	17.83 ± 10.01	visit x group	*F*_(2,57)_ = 0.24; *p* = 0.789
Testosterone (pg/mg hair) ^*GRP*^				group	*F*_(2,57)_ = 4.11; *p* = 0.002
Visit 1 (16 weeks)	0.82 ± 0.37 ^3^	0.86 ± 0.31 ^3^	1.27 ± 0.75 ^1,2^	visit	*F*_(1,57)_ = 0.01; *p* = 0.916
Visit 2 (48 weeks)	0.88 ± 0.71	0.86 ± 0.37	1.27 ± 0.64	visit x group	*F*_(2,57)_ = 0.03; *p* = 0.976

*Visit* depicts significant differences between visit 1 & 2, *GRP* depicts significant differences between groups. 1 (healthy), 2 (preeclampsia), & 3 (GDM) depict significant differences following post-hoc testing. Values are given as mean ± standard deviation. SBP: Systolic blood pressure, MAP: Mean artieral pressure, DBP: Diastolic blood pressure, SDNN: Standard deviation of the NN interval, RMSSD: Root mean the square root of the mean squared differences of successive NN intervals.

## Data Availability

Data will be made available on demand by the corresponding author via email contact.
